# Toll-like receptor 4 signaling in neurons of trigeminal ganglion contributes to nociception induced by acute pulpitis in rats

**DOI:** 10.1038/srep12549

**Published:** 2015-07-30

**Authors:** Jia-Ji Lin, Yi Du, Wen-Ke Cai, Rong Kuang, Ting Chang, Zhuo Zhang, Yong-Xiang Yang, Chao Sun, Zhu-Yi Li, Fang Kuang

**Affiliations:** 1 Department of Neurology, Tangdu Hospital, The Fourth Military Medical University, Xi’an, 710004, China; 2 Department of Neurobiology and Collaborative Innovation Center for Brain Science, School of Basic Medicine, The Fourth Military Medical University, Xi’an, 710032, China; 3 State Key Laboratory of Military Stomatology, Department of Operative Dentistry and Endodontics, School of Stomatology, The Fourth Military Medical University, Xi’an, 710032,China; 4 Department of Endodontics, Jinan Stomatological Hospital, Jinan, 250001, China; 5 Department of Cardio-Thoracic Surgery, Kunming General Hospital of Chengdu Military Region, Kunming, 650000, China

## Abstract

Pain caused by acute pulpitis (AP) is a common symptom in clinical settings. However, its underlying mechanisms have largely remained unknown. Using AP model, we demonstrated that dental injury caused severe pulp inflammation with up-regulated serum IL-1β. Assessment from head-withdrawal reflex thresholds (HWTs) and open-field test demonstrated nociceptive response at 1 day post injury. A consistent up-regulation of Toll-like receptor 4 (TLR4) in the trigeminal ganglion (TG) ipsilateral to the injured pulp was found; and downstream signaling components of TLR4, including MyD88, TRIF and NF-κB, and cytokines such as TNF-α and IL-1β, were also increased. Retrograde labeling indicated that most TLR4 positve neuron in the TG innnervated the pulp and TLR4 immunoreactivity was mainly in the medium and small neurons. Double labeling showed that the TLR4 expressing neurons in the ipsilateral TG were TRPV1 and CGRP positive, but IB4 negative. Furthermore, blocking TLR4 by eritoran (TLR4 antagonist) in TGs of the AP model significantly down-regulated MyD88, TRIF, NF-κB, TNF-α and IL-1β production and behavior of nociceptive response. Our findings suggest that TLR4 signaling in TG cells, particularly the peptidergic TRPV1 neurons, plays a key role in AP-induced nociception, and indicate that TLR4 signaling could be a potential therapeutic target for orofacial pain.

Severe dental injury leads to dental pulp inflammation known as acute pulpitis (AP) and induces a severe pulp pain^1^,^2^, which is one of the most dominant complaints of the patients. The pain intensity in AP has been anecdotally referred to as being of “the highest level possible”^1^,^2^ and in some cases the condition may progress to persistent and/or referred pain^3^,^4^,^5^. Understanding the neurobiological mechanisms involved in pulp pain is an essential prerequisite to its effective management. Trigeminal ganglion (TG) is known to be involved in pain caused by dental injury with most of its neuroinflammatory responses being similar to that observed in hyperalgesia or altered anesthesia in other inflamed tissues^2^. Neuroinflammation in TG induces neuroplasticity and neuronal sensitization which are closely linked with a series of pain-related pathological states including migraine and chronic trigeminal pain^6^. Deregulation of cytokines and/or chemokines in neuronal ganglia (such as tumor necrosis factor α (TNF-α) and interleukin (IL) 1β) triggers a cascade of events that includes the release of prostanoids and neuropeptides, and induces a change in the properties of neurons and ion channels, leading to production of other cytokines/chemokines and recruitment of macrophages^7^,^8^,^9^,^10^. These events are directly involved in the pathogenesis of allodynia and hyperalgesia. Therefore, investigating the mechanisms involved in dental injury-induced neuroinflammation of TG may help devise novel therapeutic modalities for pulp pain.

Toll-like receptor 4 (TLR4) is an important transmembrane pattern-recognition receptor of the innate immune system. TLR4 is widely expressed in the glial cells and primary sensory neurons to sense exogenous pathogen-associated molecular patterns (PAMPs) and endogenous danger-associated molecular patterns (DAMPs) released by tissues after injury or cellular stress^11^,^12^. This is well documented in inflammatory hypernociception (stimulated by complete Freund’s adjuvant, CFA), neuropathic pain (caused by spared nerve injury) and other pain related models^13^,^14^. It is also known that stimulation of TLR4 initiates a series of signaling cascades that result in the activation of nuclear factor kappa-B (NF-κB) and mitogen-activated protein kinases (MAPKs) to induce the release of pro-inflammatory cytokines such as, TNF-α and IL-1β^15^,^16^,^17^. A recent study on chronic pulpitis (CP) showed increased expression of TLR4 ligand heat shock protein (Hsp) 70 and TLR4 in TG following administration of CFA to the pulp, and blockage of TLR4 in TGs by rhodobacter sphaeroides lipopolysaccharide (LPS-RS) resulted in relief of pulp pain^18^. However, different mechanisms are involved in the causation of pain in AP and CP^2^. Whether TLR4 is also involved in the AP associated with dental injury is still not known. Moreover, the underlying mechanism of TLR4 in TG has not been adequately studied. Our study was aimed at further exploration of the neuroinflammatory mechanisms involved in the causation of pain associated with AP. The specific objectives were to: (1) establish an AP model in the rat and verify the progression of AP by pulp histology and serum cytokine detection; (2) evaluate the behavior of nociceptive response by head-withdrawal reflex thresholds (HWTs) measurement and open-field test; (3) explore the expression and signaling of TLR4 in the pulp and TGs in the AP models; and (4) observe the rescue effect of eritoran, an antagonist of TLR4, on the nociceptive response.

## Results

### Dental injury induced severe inflammation and nociceptive response at 1 day post-surgery

**Figure 1:**
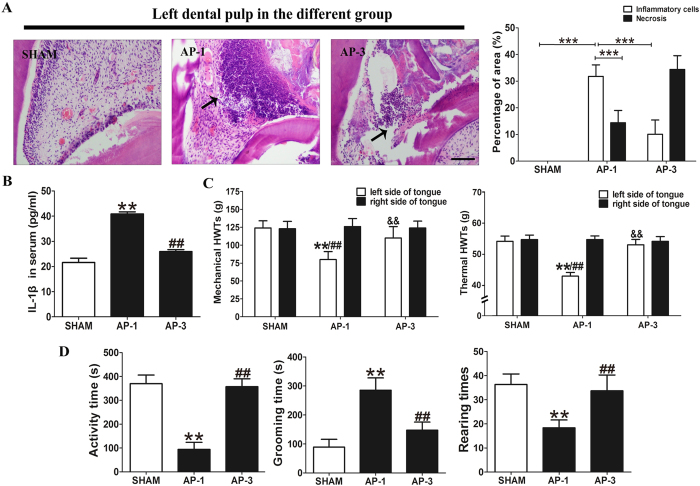
AP inflammation and behavioral change induced by dental pulp exposure. (**A**) Hematoxylin and eosin staining of left pulp at different time points of AP in rat. (Arrows indicate infiltration of inflammatory cells at the surface of necrotic and normal tissue). In SHAM group, there is normal dental pulp without inflammatory cells or necrosis; In the AP-1 group, the intensively gathered inflammatory cells occupy the most part of the dental pulp cavity with necrosis at the top; only a minimal vital pulp is left at the bottom of the cavity. In AP-3, most of the pulp tissue has developed into necrosis with few inflammatory cells inside the cavity. (Bar = 100 μm) ^***^P < 0.01; n = 8 for each group. (**B**) ELISA assay for serum IL-1βat different time points of the AP rats showing significant increase in serum IL-1β in AP-1 group as compared to that in SHAM and AP-3 groups. ^**^P < 0.01 vs. SHAM; ^##^*P* < 0.01 vs. AP-3; n = 8 for each group. (**C**) Mechanical and thermal HWTs at different time points in AP rats. There is no significant difference between the right and left TG of the SHAM and AP-3 groups. Only HWTs of the left side of tongue in AP-1 group is significantly decreased. ^**^P < 0.01 vs. left side of tongue in the SHAM group; ^##^*P* < 0.01 vs. right side of tongue in the AP-1 group; ^&&^*P* < 0.01 vs. left side of tongue in the AP-1 group; n = 8 for each group. (**D**) Results of open-field tests of AP rats at different time points. There was no significant difference between SHAM and AP-3 groups. In AP-1, spontaneous activity time was decreased, and grooming time and rearing times were significantly increased as compared to other groups. ^**^P < 0.01 vs. SHAM; ^##^*P* < 0.01 vs. AP-3; n = 8 for each group.

Left maxillary first and second molars were drilled open as the AP model, and the AP rats were assigned to AP-1 and AP-3 groups at the selected time points (1 day and 3 day post-surgery) for the following experiments. The sham operation group (SHAM group) that only received anesthesia served as the control. Hematoxylin and eosin staining and enzyme-linked immunosorbent assay (ELISA) results demonstrated that dental injury caused severe AP. Increased inflammatory cells and necrosis were observed in the exposed pulp especially at 1 day post dental injury (*P* < 0.001, Fig. 1A). There was also a significantly elevated level of inflammatory cytokine products IL-1β in the serum at 1 day post-surgery compared with the SHAM group (*P* < 0.01, Fig. 1B). Moreover, this inflammation subsided at 3 days post-injury (Fig. 1A). Fewer inflammatory cells and advancing necrosis could be seen in the pulp with relieved serum level of IL-1β compared with that at 1 day post dental injury (*P* < 0.001 and *P* < 0.01, Fig. 1A,B). These findings showed that dental injury induced a severe acute pulpitis at day 1 post-surgery. Spontaneous behavior and nocifensive reflex were recorded to assess the degree of pain caused by acute pulpitis. Compared to the SHAM group, facial grooming time was significantly increased at 1 day post model setting (*P* < 0.01, Fig. 1D), while spontaneous activity time and rearing times in AP-1 group were decreased (*P* < 0.01, Fig. 1D). Furthermore, the HWTs to mechanical and heat stimulation of the tongue ipsilateral (left) to injured pulp was also significantly decreased, compared with the right side of the tongue in the AP-1 group or the left side in the SHAM group (*P* < 0.01, Fig. 1C). Both spontaneous behavioral and nocifensive reflex of left side reversed to baseline at 3 days post pulp exposure, compared with ones in the AP-1group (*P* < 0.01, Fig. 1C,D).

### Acute pulpitis induced TLR4 up-regulation in the TG ipsilateral to inflammed pulp at day 1 post-surgery

**Figure 2:**
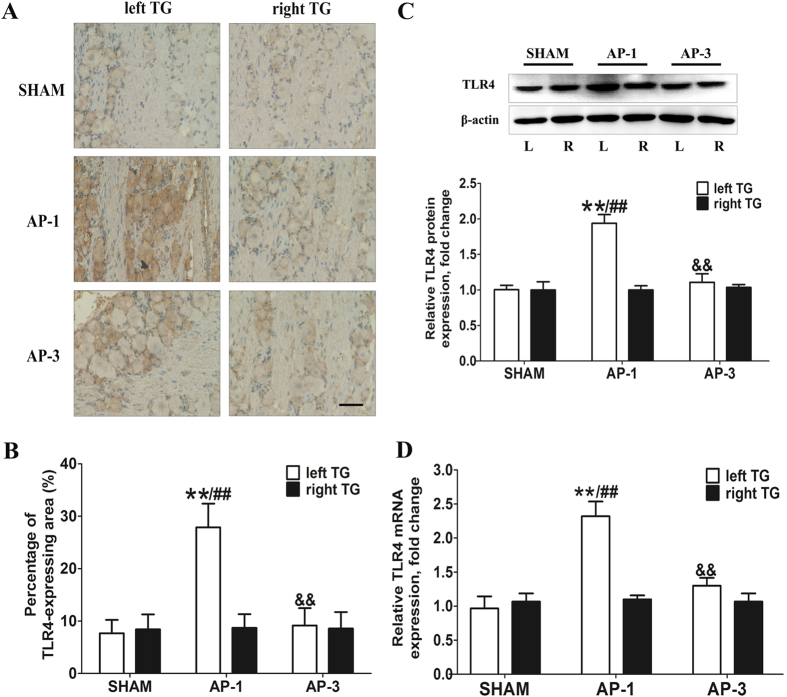
AP induced up-regulation of TLR4 expression in the TG ipsilateral (left) to the injured pulp. (**A**) and (**B**) Representative pictures of immunohistochemical labeling for TLR4 in TG at different time points of the AP rats with quantification analysis. There was no significant difference between the right and left TG of the SHAM group, and all the data are normalized by the left TG of the SHAM group. Compared to left TG of the SHAM group or right TG of the AP-1 group, the left TG of AP-1 group showed remarkable increase of TLR4 immunoreactivity. (Bar = 100 μm) ^**^P < 0.01 vs. left TG in SHAM; ^##^*P* < 0.01 vs. right TG in AP-1; ^&&^*P* < 0.01 vs. left TG in AP-1; n = 8 for each group. (**C**) Representative immunoblots of samples from rat TGs subjected to AP model with quantitative densitometric analysis of TLR4 protein with β-actin as an internal standard. The immunoblots were obtained from the microgel running in the same experiment conditions. There is no significant difference between the right and left TG of the SHAM group; the pixels of the blots are normalized by the left TG of the SHAM group for comparison. TLR4 protein level is significantly increased in the left TG in AP-1 group as compared to the other groups. ^**^P < 0.01 vs. left TG of SHAM; ^##^*P* < 0.01 vs. right TG of AP-1; ^&&^*P* < 0.01 vs. left TG of AP-1; n = 8 for each group. (**D**) Graphical representation of TLR4 mRNA expression in TGs. There is no significant difference between the right and left TG in the SHAM group, and all the data are normalized by the left TG of the SHAM group. There is a significant increase in left TG in AP-1, as compared to the other groups. ^**^P < 0.01 vs. left TG of SHAM; ^##^*P* < 0.01 vs. right TG of AP-1; ^&&^*P* < 0.01 vs. left TG of AP-1; n = 8 for each group.

Immunohistochemical labeling visualized by diaminobenzidine (DAB, Fig. 2A) showed that TLR4 immunoreactive product was light and diffused in both left and right TGs of SHAM group. However, the expression of TLR4 in the left TG was significantly up-regulated at 1 day after the upper-left-molars pulp exposure (*P* < 0.01, Fig. 2A,B), whereas there were no notable changes in the right TGs. According to the measurement of TLR4 expressing area, the microscopy showed that there was no significant change in the expression of the TLR4 of both right and left TGs in AP-3 group compared with the SHAM group. Furthermore, the results from real-time quantitative polymerase chain reaction (RT-qPCR) and western blot assay also showed that the expression of TLR4 was remarkably up-regulated in the left TGs in AP-1 group, compared with the SHAM group and the right TGs of AP-1 group (*P* < 0.01, Fig. 2C,D).

### TLR4 was up-regulated in neurons innervating the pulp and co-localized with TRPV1/CGRP in the left TG at day 1 post-AP

**Figure 3:**
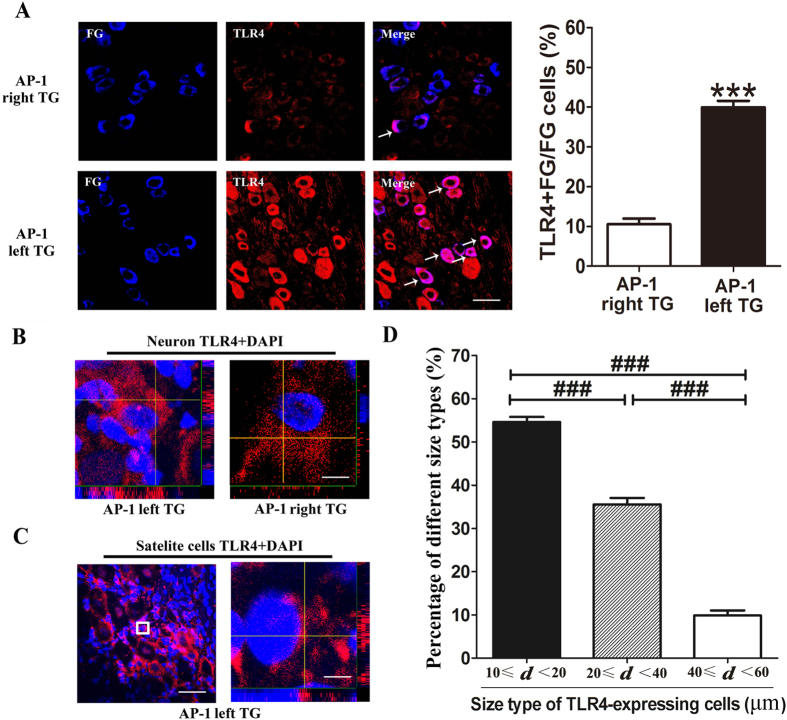
Immunofluorescent staining of TLR4 in TG at AP-1. (**A**) Retrograde labeled FG and immunofluorescent staining of TLR4 in the neurons. Representative pictures show a similar number of FG-labeled neurons in the left and right TG; but the number with TLR4 immunostaining is significantly increased in the left TG, compared to the right TG. The number of FG labeled neurons that also express TLR4 is significantly increased in the left TG compared with the right TG in the AP-1 group. ^***^P < 0.01 vs. right TG of AP-1; n = 8 for each group. (**B**) and (**C**) The stack of scanned pictures of confocal microscopy for TLR4 immunoreactivity in neurons from both TGs of AP-1 group, as well as the satellite cells. The TLR4 staining is significantly increased in the neurons of the left TG of AP-1, including cytoplasm (B section: Bar = 300 μm; C section: left: Bar = 40 μm, right: Bar = 450 μm). (**D**) Measurement of diameters of the TLR4 positive neurons. 54.59 ± 1.19% neurons are of 10–20 μm, 35.56 ± 1.49% are of 20–40 μm and 9.85 ± 1.56% are of 40–60 μm, indicating that most of the TLR4 immunoreactivity in the small and medium-sized neurons, including cytoplasm. ^###^*P* < 0.01; n = 8 for each group.

**Figure 4:**
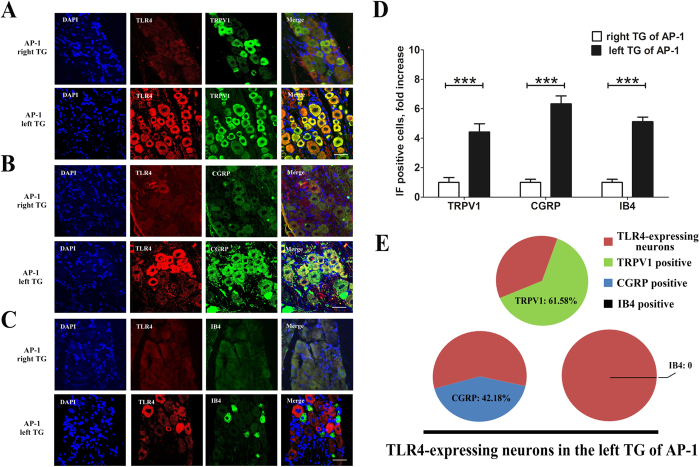
Immunofluorescence staining of TLR4 with typical pain-associated molecules. Immunofluorescent staining of TLR4 and TRPV1/CGRP/IB4 in both TGs from the AP-1 group with quantification analysis. The results showed that the number of TRPV1/CGRP/IB4 cells was significantly increased in the left TG, compared to the right TG of the AP-1 group. The double labeled staining revealed that increased expression of TLR4 was mainly in the TRPV1- or CGRP- positive neurons, but not in IB4-positive neurons of the left TGs of the AP-1 group (Bar = 40 μm). ^***^P < 0.01; n = 8 for each group.

In order to confirm the innervation of pulp by TG neurons, retrograde labeling with fluorogold (FG) was done. The number of FG labeled neurons that also expressed TLR4 was significantly increased in the left TG as compared to the right TG in the AP-1 group (*P* < 0.001, Fig. 3A). Measurement of diameters of the TLR4 positive neurons showed that 54.59 ± 1.19% neurons were of 10–20 μm, 35.56 ± 1.49% were of 20-40 μm and 9.85 ± 1.56% were of 40–60 μm (Fig. 3D). These data indicated that most of the TLR4 immunoreactive products were mainly in the small and medium-sized neurons, including that in the cytoplasm (Fig. 3B). The TLR4 staining was also observed in the satellite cells of both TGs, but we did not discern any significant difference between the right and the left (Fig. 3C). Furthermore, immunofluorescent staining showed that the numbers of transient receptor potential vanilloid 1 (TRPV1), calcitonin gene-related peptide (CGRP) and isolectin B4 (IB4) positive cells were significantly increased in the left TG, as compared to the right TG of the AP-1 group (Fig. 4A–D). Meanwhile, the double labelled staining showed that the increased expression of TLR4 was mainly in the TRPV1 or CGRP-positive neurons, but not in IB4-positive neurons in the left TGs of the AP-1 group (Fig. 4E).

### AP induced up-regulated TLR4 signaling expression in the TG ipsilateral to inflammatory pulp

**Figure 5:**
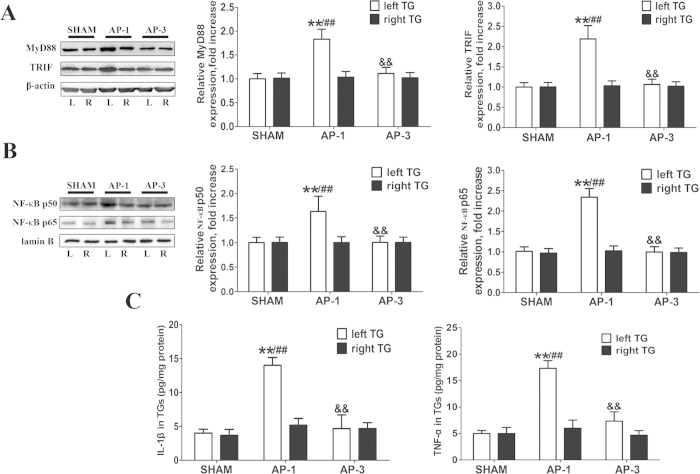
Expression of downstream molecules of TLR4 in the TGs. (**A**) and (**B**) Representative immunoblots of samples from TGs of AP rat with quantitative densitometric analysis with β-actin/lamin B as an internal standard. The immunoblots were obtained from the microgel running in the same experimental conditions. There is no significant difference between the right and left TG of the SHAM group, and the pixels of the blots are normalized by the left TG of the SHAM group for comparison. The statistical analysis indicated that MyD88, TRIF and NF-κB (in the nucleus) protein levels were significantly increased in left TG of the AP-1 group, compared to the other groups. ^**^P < 0.01 vs. left TG of SHAM; ^##^*P* < 0.01 vs. right TG of AP-1; ^&&^*P* < 0.01 vs. left TG of AP-1; n = 8 for each group. (**C**) Results of ELISA assay for IL-1β and TNF-α in the TGs at different time points of AP rats: There was no significant difference between the right and left TG of the SHAM group, and the data were normalized by the left TG of the SHAM group. The statistical analysis indicated that IL-1β and TNF-α are significantly increased in left TG of the AP-1 group, as compared to that in the other groups. ^**^P < 0.01 vs. left TG of SHAM; ^##^*P* < 0.01 vs. right TG of AP-1; ^&&^*P* < 0.01 vs. left TG of AP-1; n = 8 for each group.

Downstream transcriptional signaling of TLR4 was examined. The western blot assay showed that the levels of myeloid differentiation primary response gene 88 (MyD88) and TIR domain-containing adaptor inducing interferon-β (TRIF) were significantly up-regulated in the left TG of the AP-1 group when compared with the right-sided or baseline expression (*P* < 0.01, Fig. 5A), while there was no significant difference between the two sides of TGs in the protein levels of the SHAM or AP-3 group (Fig. 5A). Furthermore, the nuclear NF-κB p65 and p50 were also significantly increased in the left TGs of the AP-1 group (*P* < 0.01, Fig. 5B). The ELISA results showed that the expression levels of TNF-α and IL-1β were elevated in the left TG of AP rat at 1 day post-surgery (*P* < 0.01, Fig. 5C). At 3 days of AP, these down-stream transcriptional signals of TLR4 as well as the cytokines dropped back to normal levels.

### TLR4 blockade of TG by eritoran relieved nociceptive response in AP

**Figure 6:**
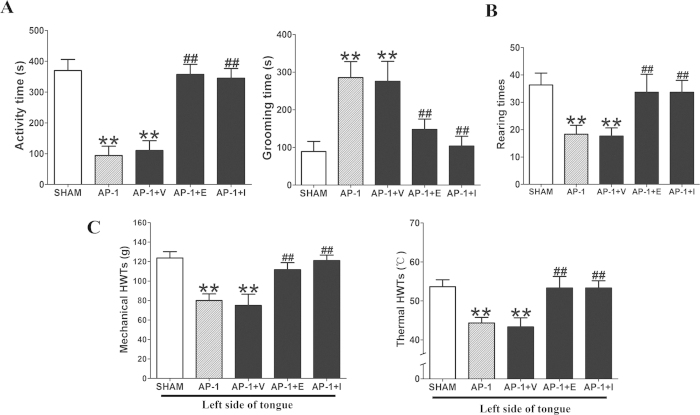
Effect of eritoran on behavior of nociceptive response at 1 day post-pulp exposure. Results from the open-field test and HWTs test in different groups: compared to the other groups, the nociceptive response was significantly increased in left TG in AP-1 group, which was rescued by eritoran or ibuprofen administration. After eritoran or ibuprofen administration, the activity time was increased significantly while the grooming time was down-regulated in the AP-1 + E group compared with the AP-1 group. The rearing times were also increased in the AP-1 + E and AP-1 + I group compared with the AP-1 group. The level of the HWTs to mechanical and heat stimulation of the tongue ipsilateral to the injured pulp were significantly higher in the AP-1 + E/AP-1 + I group than AP-1 group, while it showed no difference between the AP-1 and AP-1 + V group. ^**^P < 0.01 vs. SHAM; ^##^*P* < 0.01 vs. AP-1; n = 8 for each group.

To explore the role of TLR4 in pulp pain of AP, the AP-1 rats received eritoran (selective TLR4 inhibitor, E), ibuprofen (analgesic, I) and vehicle (0.9% NaCl solution, V); and were categorized as AP-1 + E group, AP-1 + I group and AP-1 + V group, respectively. After eritoran and ibuprofen administration, the activity time was increased significantly (*P* < 0.01, Fig. 6A) while the grooming time was down-regulated in the AP-1 + E group compared with the AP-1 group (*P* < 0.01, Fig. 6A). The rearing times were also increased in the AP-1 + E and AP-1 + I groups when compared with the AP-1 group (*P* < 0.01, Fig. 6B). However, there were no statistically significant differences in the nociceptive response between the AP-1 and AP-1 + V groups (Fig. 6A,B). Furthermore, the levels of the HWTs to mechanical and heat stimulation of the tongue ipsilaterally to injured pulp were significantly higher in the AP-1 + E and AP-1 + I group than AP-1 group (*P* < 0.01, Fig. 6C), while no difference was found between the AP-1 and AP-1 + V group (Fig. 6C).

### TLR4 blockade by eritoran in left TG restrained up-regulated downstream molecules subject to AP

**Figure 7:**
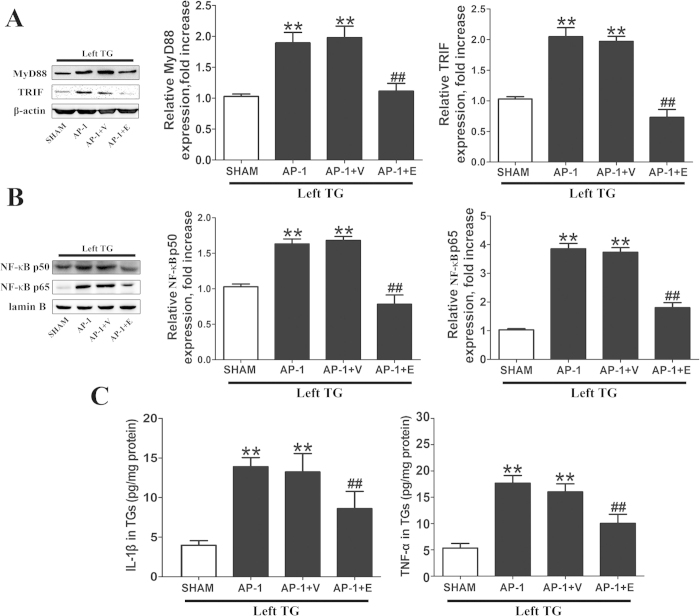
Effect of eritoran on the expression of downstream molecules of TLR4 in left TG. (**A**) and (**B**) Representative immunoblots of samples from left TGs from different groups with quantitative densitometric analysis with β-actin/lamin B as an internal standard. The immunoblots were obtained from the microgel running in the same experimental conditions. There was no significant difference between the right and left TG of the SHAM group, and the pixels of the blots were normalized by the left TG of the SHAM group for comparison. Compared to the other groups, MyD88, TRIF and NF-κB (in the nucleus) protein levels were significantly increased in left TG of the AP-1 group, which were rescued by the eritoran.^**^P < 0.01 vs. SHAM; ^##^*P* < 0.01 vs. AP-1; n = 8 for each group. (**C**) ELISA assay for IL-1β and TNF-α in left TGs from different groups. Compared to the other groups, there was a significant increase in left TG of AP-1 rats, which was rescued by the eritoran. ^**^P < 0.01 vs. SHAM; ^##^*P* < 0.01 vs. AP-1; n = 8 for each group.

After eritoran administration into TGs, the left TGs from different groups were harvested for examination. The western blot and ELISA assay showed that MyD88, TRIF, NF-κB p65 and p50, TNF-α and IL-1β in the left TGs of AP-1 + E group were significantly decreased as compared to that in the AP-1 group (*P* < 0.01, Fig. 7). However, these expression levels in both AP-1 and AP-1 + V group were still higher than that in the SHAM group (*P* < 0.01, Fig. 7), and there were no statistically significant differences between the AP-1 and AP-1 + V group.

## Discussion

### AP induces a transient nociceptive response at the initiation of pulp inflammation as compared to the relatively longer nociceptive response in CP

The model we used in the present study is a typical pulpitis model provoked by type III dental injury which destroys most part of the pulp^2^,^19^. In this model, the biological process includes mechanical injury, inflammation, necrosis of the pulp and nociceptive response. As shown by histological observation of the dental pulp, there was a dramatic increase in inflammatory cells that had invaded the central part of the pulp, with part of the vital pulp at the bottom and with necrotic tissue at the top at 1 day post-surgery. The model captures progression of both inflammation and necrosis from immediately after the dental injury. Although we cannot exclude the bacterial and mechanical stimulation of the TG in our study, this model indeed evokes nociceptive response and has been widely used for pain research in stomatology^2^,^20^. In our study, immunohistochemistry exhibited that the typical pain-associated molecules (TRPV1, CGRP and IB4) were significantly up-regulated in the ipsilateral TG^21^,^22^. Meanwhile, the change observed in the behavior tests (HWTs and open field test) also demonstrated nociception, which was rescued by the traditional analgesic ibuprofen. All these changes indicate that the model captured nociception induced by AP. Although behavioral analysis is the most common method to measure nociceptive response, it is hard to apply it directly to measure orofacial nociception. Experimental animals are always evasive when they perceive foreign matters in von-frey filaments test and heat probe test. The results of weight gaining and sucrose solution consumption may also be influenced by eating disturbances induced by orofacial inflammation and food intake^20^. In contrast, HWTs measurement and open-field test described above are more suitable for assessing nociceptive response. Trigeminal ganglion (TG) neurons innervate both pulp and tongue, and this anatomical feature favors the development of tongue (referred) pain associated with pulp inflammation^18^. Therefore, tongue-referred pain could be used to measure pulp pain caused by injury/inflammation. In addition, open-field test is widely used in psychoneurosis analysis^23^, which could also reflect characteristic emotional change caused by orofacial pain^24^,^25^. Reduced activity time and exploratory behaviors (rearing times) indicate stress and anxiety component within spontaneous activity^20^,^26^. Excessive facial grooming is a characteristic behavior indicative of orofacial pain^27^. In the present study, all behavioral assessments demonstrated severe nociception evoked by AP at 1 day post-surgery. The consistency between peak nociceptive response and the peak of AP suggests a close link between them. Compared to nearly 5 days of advancing inflammation in the pulp, the behavioral tests showed significant changes only at 1 day post-surgery, though the tendency of relieved nociception continued. This observation is consistent with the clinical finding that pulpitis might induce severe pain until pulp necrosis, as advancing necrosis usually relieves the pain^1^. In the present study, most of the pulp developed into necrosis at 3 days post-surgery, which is a typical result from type III dental injury. At 3 days post-surgery, there was still but only little vital pulp present which decreased the afferent nerve activity. Furthermore, pulpitis may proceed to total pulp necrosis with only minor pain, or without any symptoms, probably because of strong local regulatory mechanisms^2^.

### Increased expression of TLR4 in TG is involved in the nociception associated with AP in the rat

At the same time point as the rats apparently exhibited the peak nociceptive response, a significant increase of TLR4 expression was found in the TG ipsilateral to the dental injury. Although TLR4 sparked great interest in therapeutic manipulation of the innate immune system with the identification of the bacterial endotoxin receptors^28^, this receptor was also extensively expressed on glial cell and certain small primary afferent neurons^11^,^29^. Expression of TLR4 in the primary sensory neurons of dorsal root ganglion (DRG) and TG have rarely been reported except in the field of pain research. Earlier studies have demonstrated that TLR4 expressed in the neurons of the DRGs played a key role in pain caused by tissue injury or inflammation; and blockade of TLR4 signaling reduced hyperalgesia^30^,^31^. Furthermore, the number of TLR4-immunoreactive neurons was significantly larger compared with control during CFA-mediated chronic pulp pain or pain due to odontogenic infection^18^. Here we provided direct evidence that AP induced by dental injury also led to a significant up-regulation of TLR4 expression in TGs. The numbers of TLR4-immunoreactive neurons was significantly increased in the TG ipsilateral to dental injury. Although TLR4 is functionally located in the cell membrane, our study showed that the increased TLR4 expression was also found in the cytoplasm, mainly in the small- and medium-sized neurons. Interestingly, consistent with the nociceptive response of AP, this increase of TLR4 expression was transient and mainly observed in the initial stage of pulp inflammation. After blockade of TLR4 by its antagonist, eritoran, the nociceptive response was significantly inhibited. All these findings suggest that increased TLR4 be a key molecule in mediation of nociceptive response caused by AP.

TRPV1 is mainly expressed in sensory neurons that detect noxious stimuli, and has been extensively linked with painful and inflammatory processes^32^. Increased expression of TRPV1 in TG is involved in various kinds of orofacial pain^33^,^34^,^35^. In our study, immunofluorescent staining showed that the number of TRPV1 positive neurons was significantly increased in the TG ipsilateral to dental injury, which also indicated a nociceptive response in the AP rat. This is consistent with a previous study showing that TLR4 was mainly expressed in the TRPV1 positive neurons of normal TG^36^. The increase of TLR4 in TG of AP rat in our study was also mainly in the TRPV1 positive neurons. Further exploration showed that the neurons with high expression of TLR4 were mainly CGRP positive but not IB4 positive. The co-localization of TLR4 with CGRP-containing neurons in our study indicated that peptidergic nociceptive fibres reacted to AP-induced nociceptive response, which was consistent with the findings from another study showing pain-mediated sensitization of nociceptors in DRG^37^. However, compared with TLR4 expression in the DRG of pain models^37^, the TLR4-expressing neurons in TG did not show IB4 positive staining in AP rat. Although projection of non-peptidergic afferents to rodent tooth pulp was also involved in the tooth pain signal transduction^38^, the previous study showed that IB4 positive nociceptors were mainly involved in persistent pain^39^. Considering the transient nociceptive response of AP rats, the up-regulation of TLR4 in peptidergic sensory neurons but not in non-peptidergic sensory neurons indicates a response to the transient pain. Additionally, we found that TLR4-immunoreactive staining also existed in the satellite cells in both sides of TGs in the AP-1 rats. Since most satellite cells enclosed the neurons in TG and the intense staining of TLR4 in neurons could have covered the TLR4 staining of satellite cells in AP rat, we could not ascertain for sure whether the TLR4 expression was also increased in these satellite cells. Although TLR4 expression in satellite cells usually plays a role in innate immune reaction to DAMPs and PAMPs^11^,^12^, it has also been widely reported that TLR4 in the satellite cell-modulated neuronal excitation initiated neuroinflammatory responses, and contributed to the initiation of neuropathic pain^40^,^41^. Furthermore, there is evidence that TLR4 is involved in the crosstalk between primary sensory neurons and satellite cells^42^, and that various pathological conditions of the oral-mandibular system induce activation of satellite cells^43^. Therefore, we propose that the TLR4 expression in the satellite cells of TG may also be involved in the AP-induced innate immune reactions, which ultimately contributed to the transmission of the nociceptive signals.

### Both MyD88-dependent and MyD88-independent signaling pathways are involved in the neuroinflammation induced by TLR4 in the TG of AP

TLR4 signaling is known to be activated by the ligation of PAMPs and DAMPs^11^,^12^. However, it is known that the TLR4 in the soma of neurons in TGs is not directly involved in pulp inflammation or infection in AP rats. The activation and up-regulation of TLR4 in left TG may be due to the stimulation to trigeminal nerve and DAMPs released after tissue injury or cellular stress of dental inflammation^11^,^18^. TLR4 in the neurons may also reflect an adaptive phenomenon in response to the external stimuli, acting as a security and alarm system for negative factors, akin to the mechanism associated with glia of the central nervous system (CNS)^12^. Although the increase in TLR4 expression in the CP model is well documented^18^, the precise role of this receptor in regulation of orofacial pain is not known. Earlier studies have shown that activation of TLR4 usually triggers two classic signaling cascades: MyD88-dependent and/or MyD88-independent pathway, which involves initial recruitment of MyD88 and/or TRIF. Propagation of both these signals subsequently result in the translocation of NF-κB to the nucleus that leads to the production of pro-inflammatory cytokines^44^,^45^,^46^. In our study we showed that AP promoted activation of both downstream signaling pathways of TLR4, and increased cytokines TNF-α and IL-1β in TG. Blockade of TLR4 inhibited the release of these cytokines. These findings point towards a TLR4-induced neuroinflammation in TG in the AP model setting. Increased expression of TNF-α and IL-1β is known to produce a variety of effects that mediate pain in the formalin model, chronic constriction injury (CCI) model, chronic compression of dorsal root (CCD) model and other pain related models. For example, the direct application of TNF-α enhances tetrodotoxin-resistant (TTX-R) Na^+^ channels and increases membrane K^+^ conductance by a non-voltage-gated mechanism^47^,^48^, triggering production of cytokines and inducing cell death^49^,^50^. Moreover, IL-1β is known to directly cause the up-regulation of pro-nociceptive mediators and induce complex signaling cascades, thus potentiating heat-activated excitatory inward currents^51^. Both TNF-α and IL-1β lead to neuronal excitability and sensitization^51^,^52^,^53^, which ultimately induces thermal hyperalgesia and mechanical allodynia^51^,^54^,^55^,^56^. Inhibition of IL-1β or TNF-α can reduce pain symptoms and pain behaviors as observed in various pain models in rodents and human subjects^57^,^58^,^59^,^60^,^61^. Therefore, the TLR4-mediated neuroinflammation in TG of AP rat causes the severe nociception at 1 day post dental injury, and blockage of TLR4 displays an ability to control this neuroinflammation and pain, indicating that TLR4 in TG may be a potential therapeutic target for orofacial pain.

## Methods

### Animals and AP model

Adult male Sprague-Dawley rats weighing 250 to 350 g used in the present study were obtained from the experimental animal center of the Fourth Military Medical University. The animals were maintained in a temperature-controlled room (23 °C) with a 12-hour light/dark cycle. Food and water were made freely available. All the experimental procedures were approved by the Fourth Military Medical University Committee on Animal Care and Use. The study was conducted in accordance with the guidelines of the International Association for the Study of Pain. The AP model was established as described^62^. The rats were lightly anesthetized with 2% isoflurane in oxygen and then deeply anesthetized with an intraperitoneal application of 10% chloral hydrate (1 mL/250 g body weight). Then, the rats were placed on a warm mat (37 °C) in the supine position to perform the surgical procedure. The rat’s mouth was gently opened with metal tweezers and the left maxillary first and second molars were drilled open by a low-speed dental drill with a round tungsten carbide bur under water cooling. The whole enamel and dentin on occlusal surfaces were grinded off to induce type III injury and subsequent irreversible pulpitis with the exposure of pulp to the oral environment^2^. Then the exposed pulp was kept open and rats were conveyed back. The SHAM animals were subjected to the same process of anesthesia but without any other intervention.

### Drug application

In order to administer TLR4 antagonist eritoran into TG, the rats were anesthetized, and the skull was exposed and a small hole (1 mm in diameter) was drilled above the maxillary division of the left TG 6.5 mm anterior to interaural 0 and 2.3 mm lateral to the midline^18^. The guide cannula was extended into the hole 9 mm below the skull surface to reach TG and was fixed to the skull with two stainless-steel screws and dental quick cure resin, using the method described by Kinuyo^18^. The guide cannula were installed one week before the AP model setting. When the dental pulp were drilled open, the rats were administered vehicle (0.5 μL/day, 0.9% NaCl solution) or TLR4 antagonist eritoran (0.1 mM, 0.5 μL/day; Eisai) into TG once a day for a total of three successive days. Ibuprofen (3.0 mg/kg) was orally administered 60 min prior to the behavior test.

### Open-field test

All behavioral tests were conducted during the light phase of the light/dark cycle. The tests were performed 1 day and 3 day post-surgery. The rats were brought to the test room one by one for behavioral testing and returned to the colony room immediately afterwards. In all tests, the animal’s response was recorded on an overhead video tracking system (Plexon) which was positioned vertically 2 metres above the test field. The results were later analyzed by an observer blinded to the animals’ group assignment. The open-field test was performed to seek out any sign of motor impairment that could have been caused by surgery or eritoran administration^24^,^25^,^63^. Rats were placed at one corner of a 70 × 70 cm open field surrounded by 30 cm high cardboard walls. The floor was made of plexiglas. The animals’ behavior in the open field was recorded for 30 min for each time point. The following parameters were analyzed: (1) activity time (seconds of movement during testing), (2) facial grooming time and (3) rearing times. Time was measured with a stopwatch and rearing times were counted manually.

### Head-withdrawal reflex thresholds (HWTs) measurement

HWT is a test to check the response to mechanical and heat stimulation to the lateral edge of tongue (3 mm posterior from tip of tongue). It was measured under light anesthesia with 2% isoflurane in oxygen at the scheduled timepoint as described above^18^. Bipolar enamel-coated stainless steel wire electrodes (Narishige) were placed in the splenius capitis muscle for electromyogram (EMG) recording of the reflex response (inter-electrode distance, 5–6 mm). The jaw of the rat was gently pulled with plastic strings and the rat’s mouth was kept open, and then mechanical stimulation (0–130 g) was applied to the lateral edge of the tongue ipsilateral to the exposed pulp, using the forceps with flat tips to the lightly anesthetized rats. The stimulus velocity was manually controlled consecutively from 0 g to threshold values at a speed of approximately 10 g/s. The threshold intensity for evoking EMG activity by mechanical stimulation of the tongue was defined as the mechanical HWT. Heat stimulation (35–60 °C) was also applied to the lateral edge of the tongue ipsilateral to the exposed pulp, using a contact heat probe (Intercross) in the lightly anesthetized rats. The threshold temperature for evoking EMG activity by heat stimulation to the tongue was defined as the heat HWT. The mechanical or heat stimulation was applied three times with five-minute intervals, and the mean value of the HWTs was calculated.

### Hematoxylin and eosin staining of dental pulp

The rats were decapitated at selected time points (1 day and 3 day) and the molar teeth specimens were rapidly removed and cut into fragments subjected to decalcification in ethylenediaminetetraacetic acid (EDTA) (41.3 g disodium EDTA, 4.4 g NaOH in 1000 mL distilled water) solution for 30 days, then fixed with 4% paraformaldehyde for 48 h. Next specimens were embedded in paraffin wax and cooled at 2–8 °C. The embedded specimens were sliced with a rotary microtome to yield slices with a thickness of 4 μm. The microtomed slices were mounted onto microscopic glass slides, and treated with 50–60 °C water. After being tempered overnight in an oven maintained at 37 °C, the slices were stained with hematoxylin and eosin, sealed with fat-soluble gel and examined microscopically.

### Immunohistochemistry staining

The rats were lightly anesthetized with 2% isoflurane in oxygen and then deeply anesthetized with an intraperitoneal application of 10% chloral hydrate (1 mL/250 g body weight) under anesthesia. Then the animals were perfused intracardiac with phosphate buffer solution (PBS) containing heparin and followed with 4% paraformaldehyde in PBS (pH 7.4) as fixative for 10 minutes. The TGs specimens were rapidly removed and post fixed overnight at 4 °C, and then were directly dehydrated in graded alcohol and processed for embedding in paraffin wax with the anatomical orientation preserved. Sections 3–4 μm thick were cut according to standard procedures, mounted on silane-coated slides, and finally air-dried. The sections were placed in a bathing solution of 3% H_2_O_2_ and 60% methanol PBS (pH 7.4) for 30 min and then treated with 0.01 mol/L sodium citrate buffer at 95 °C in a microwave oven for 13 min (antigen retrieval). Thereafter, specimens were treated with 5% normal goat serum and 5% bovine serum albumin in PBS. Before each step, sections were rinsed three times in PBS buffer. For DAB staining of TLR4, sections rinsed with PBS were incubated with the TLR4 primary antibody (mouse anti-TLR4: Abcam, ab30667, 1:150) overnight, and then corresponding secondary biotinylated goat anti-mouse antibodies for 1 h at room temperature (goat anti-mouse IgG: Abcam, ab6788, 1:400). Then streptavidin (Abcam, ab7403 1:10000) were used to incubate the sections for 1 h. Finally, staining was developed with 3,3′diaminobenzidine tetra-hydrochloride in 0.05 mol/L Tris-HCl buffer and 0.1% H_2_O_2_, and examined microscopically. For immunofluorescent doublelabelled-staining, the expression of TLR4, TRPV1 and CGRP was examined by primary antibody (mouse anti-TLR4: Abcam, ab30667,1:150; rabbit anti-TRPV1: Abcam, ab63083, 1:150; rabbit anti-CGRP: Millipore, AB5920,1:2000) overnight, and were then detected by anti-mouse-conjugated AlexaFluor594 (Abcam, ab150116, 1:1000) and anti-rabbit-conjugated fluorescein isothiocyanate (FITC) (Abcam, ab6717, 1:1000) with 4′,6-diamidino-2-phenylindoledihydrochloride (DAPI, Santa Cruz, CAS 28718-90-3, 1:2000) staining for the nucleus. The IB4 were detected just with fluorochromes with FITC (FITC-IB4: Vector Laboratories, FL-1201, 1:200).

### Retrogratelabeling with FG tracer

The rats were lightly anesthetized with 2% isoflurane in oxygen and then deeply anesthetized with an intraperitoneal application of 10% chloral hydrate (1 mL/250 g body weight). Then, the rats were placed on a warm mat (37 °C) in the supine position to perform the surgical procedure. The rat’s mouth was gently opened with metal tweezers and the left maxillary first and second molars were drilled open by a low-speed dental drill with a round tungsten carbide bur under water cooling. Five μL of 10% FG (Fluorochrome) dissolved in saline was applied into the dental pulp. Then the holes were sealed by hydraulic temporary restorative (G.C. Corporation). After recovery from anesthesia, the rats were subjected to behavior tests. The rats not demonstrating the typical behavior associated with nociceptive response, were selected for establishing AP model in 3 days. The SHAM animals were subjected to the same process of anesthesia but without any other intervention.

### Image analysis

For DAB staining, the images of immunohistochemical results were obtained by a DMR-X microscope coupled with a DC500 digital camera (Leica) and the image analysis system Quantimet Q550 (Leica). Nine randomly selected discontinuous fields (20x) per samples were evaluated. The positive area and total area of TLR4 immunostaining were obtained and quantified by Image-Pro Plus software (Media Cybernetics). The percentage of expression area (PEA) was measured as (positive area)/(total area) × 100%.

For immunofluorescent double labelled staining, the images were collected by a Leica SP5 confocal microscope (Leica) and recorded sequentially using Leica Application Suite Software (Leica). Nine randomly selected discontinuous fields (20x) per samples were evaluated. The counterstained sections were used for measurement of cell soma diameters. Cell soma diameters of TG neurons were measured under the microscope. The average diameters were calculated by averaging the major diameter with the minor diameter; the major and minor diameters were the longest and shortest axes through the nucleolus, respectively. For the retrograde labelling, the images were collected by a Leica SP5 confocal microscope (Leica) and recorded sequentially using Leica Application Suite Software (Leica). Nine randomly selected discontinuous fields (20x) per samples were evaluated. The number of FG positive neurons in TG were counted, and the TLR4 immunoreative neurons among them were calculated by the following formula: (the number of neurons dually-labeled with TLR4 and FG)/(the number of neurons labeled with FG) × 100%.

### RT-qPCR assay

Both TGs were rapidly removed and promptly frozen on dry ice after decapitation of rats. RT-qPCR assay of TLR4 mRNA expression was performed using RNAs isolated from TGs of the rats. The tissue total RNA was extracted using Trizol reagent according to the manufacturer’s instructions (Takara). RNA was transcribed to cDNA with PrimeScript RT reagent Kit (Takara). Then RT-qPCR was performed using the CFX96TM real-time system (Bio-Rad), and the relative gene expression was normalized to internal control β-actin. The analysis of the melting curve of each amplified PCR product and the visualization of the PCR amplicons on 1.5% agarose gels allowed control of the specificity of the amplification. Primer sequences for SYBR Green probes of target genes are as follows: TLR4-f: GGCATCATCTT-CATTGTCCTTG, TLR4-r: AGCATTGTCCTCCCACTCG; β-actin-f: GGAGATTACTGCCCTGGCTCCTA, β-actin-r: GACTCATCGTACTCCTGCTTGCTG.

### Western blotting assay

After pretreatment, the rats from different groups underwent decapitation and both TGs were rapidly removed and quickly frozen on dry ice. Proteins were extracted from TG tissue lysates. For nuclear protein extraction, the suspension was mixed with detergent and centrifuged for 30 s at 14,000 g. The nuclear pellet was resuspended in complete lysis buffer in the presence of protease inhibitor cocktail. For the total protein extraction, the cell suspension from different groups was directly lysed by incubation for 30 min with lysis buffer and protease inhibitors on ice (Sigma). Both the nuclear protein lysates and total protein lysates were centrifuged at 12,000 r/min for 10 min to remove insoluble material. The protein concentration was determined using a bicinchoninic acid (BCA) kit (Sigma). The same amount of protein (80 μg) was used for western blot analysis. The samples were resolved in 10% SDS-PAGE gels and transferred to polyvinylidene fluoride membranes. The immunoblots were probed with anti-TLR4 (Abcam, ab13556, 1:500), anti-TRIF (Abcam, ab101232, 1:250), anti-MyD88 (Abcam, ab2064, 1:500), anti-NF-κB (p65) (Abcam, ab32536, 1:5000), anti-NF-κB (p50) (Abcam, ab32360, 1:1000) antibodies overnight at 4 °C followed by incubation with goat anti-rabbit IgG (Abcam, ab32360, 1:2000) at room temperature for 1 h. The blots were visualized using ECL-Plus reagent (Millipore). For nuclear protein, lamin B (anti-lamin B: Abcam, ab16048, 1:500) was used as a loading control. For total protein, β-actin (anti-β-actin: Abcam, ab179467, 1:5000) was used as a loading control.

### ELISA

To detect the cytokines levels, blood samples were collected from rats in test tubes without anti-coagulant under sterile conditions. Serum was separated by centrifugation at 1500 r/min for 10 min. Samples were divided in small aliquots and stored at −80 ^o^C for future cytokine assessment. Then rats were subjected to decapitation and both TGs were rapidly removed and quickly frozen on dry ice. Samples were homogenized in PBS buffer by mechanical trituration method and centrifuged at 20,000 r/min for 30 min at 4 °C. Protein concentration in the supernatant was determined by BCA protein assay as per manufacturer’s instructions (Sigma). IL-1β and TNF-α concentrations were determined by commercially available ELISA kit according to the manufacturer’s protocol (Sigma). Each cytokine sample was run in duplicate and the mean cytokine concentration was calculated.

### Statistical analysis

All data were expressed as mean ± SEM. Data associated with both TGs were normalized by the right side of SHAM group for each experiment, for there is no statistical difference between the right/left TG in the SHAM group and the right TG in the AP-1 group. Statistical analyses were performed by Student’s *t*-test, one-way analysis of variance (ANOVA) or two-way repeated-measures ANOVA followed by Bonferroni’s multiple comparison tests where appropriate. A value of *P* < 0.05 was considered as significant.

## Additional Information

**How to cite this article**: Lin, J.-J. *et al*. Toll-like receptor 4 signaling in neurons of trigeminal ganglion contributes to nociception induced by acute pulpitis in rats. *Sci. Rep*. **5**, 12549; doi: 10.1038/srep12549 (2015).

## Supplementary Material

Supplementary Information
