# Supplementary material for: Toll-like receptor 4 signaling in neurons of trigeminal ganglion contributes to nociception induced by acute pulpitis in rats
Source: Sci Rep. 2015 Jul 30;5:12549. doi: 10.1038/srep12549 (PMC4519790; doi:10.1038/srep12549)
Supplement: Supplementary Information [file srep12549-s1.pdf]

Supplementary materials

For *Toll-like receptor 4 signaling in neurons of trigeminal ganglion contributes to pain induced by acute pulpitis in rats*

Jia-Ji Lin [1,2*], Yi Du [3,4*], Wen-Ke Cai [5,1*], Rong Kuang [3*], Ting Chang [1], Zhuo Zhang [1], Yong-Xiang Yang [1], Chao Sun [1], Zhu-Yi Li [1#], Fang Kuang [2#]

[1]Department of Neurology, Tangdu Hospital, The Fourth Military Medical University, Xi'an, Shannxi, 710004, China.

[2]Institute of Neurosciences, The Fourth Military Medical University, Xi'an, Shannxi, 710032, China.

[3]State Key Laboratory of Military Stomatology, Department of Operative Dentistry and Endodontics,School of Stomatology, The Fourth Military Medical University, Xi'an, Shannxi, 710032, China.

[4]Department of Endodontics, Jinan Stomatological Hospital , Jinan, Shandong, 250001, China.

[5]Department of Cardio-Thoracic Surgery, Kunming General Hospital of Chengdu Military Region, Kunming, Yunnan, 650000, China.

* The authors contributed equally to this work.

[#] Corresponding author.

Figure s1:

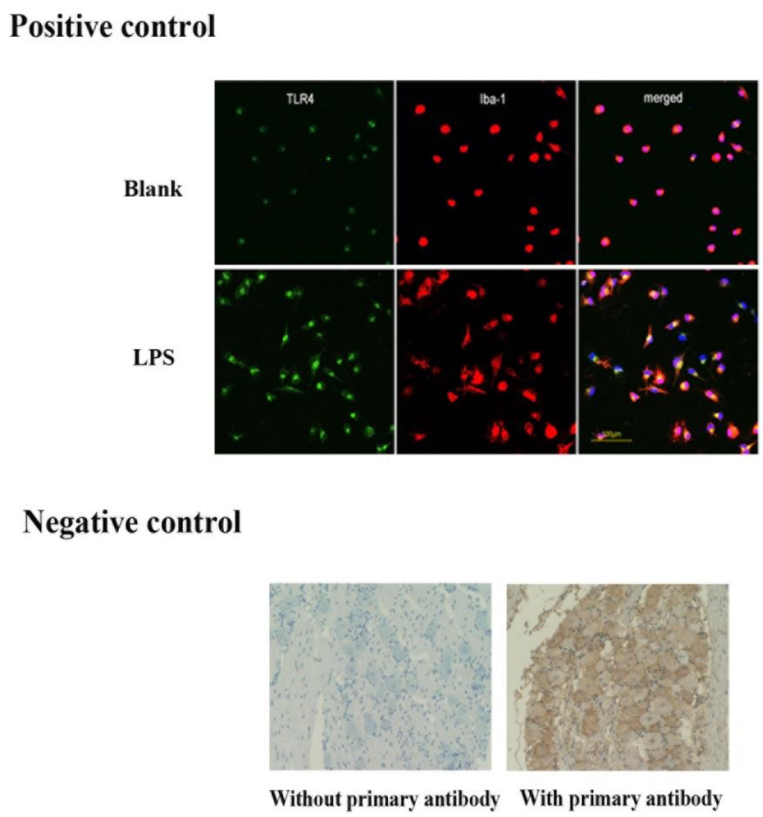

Figure s1 Positive and negative controls for TLR4 antibody. Positive control: increased expression of TLR4 were found in cultured N9 microglia stimulated by LPS, compared with blank. The microglial cells were marked with the Iba-1 immunolabeling. Negative control: the sections of left TG of AP-1 were incubated with/without the primary antibody in the DAB staining with haemotoxylin for nucleus.

Figure s2:

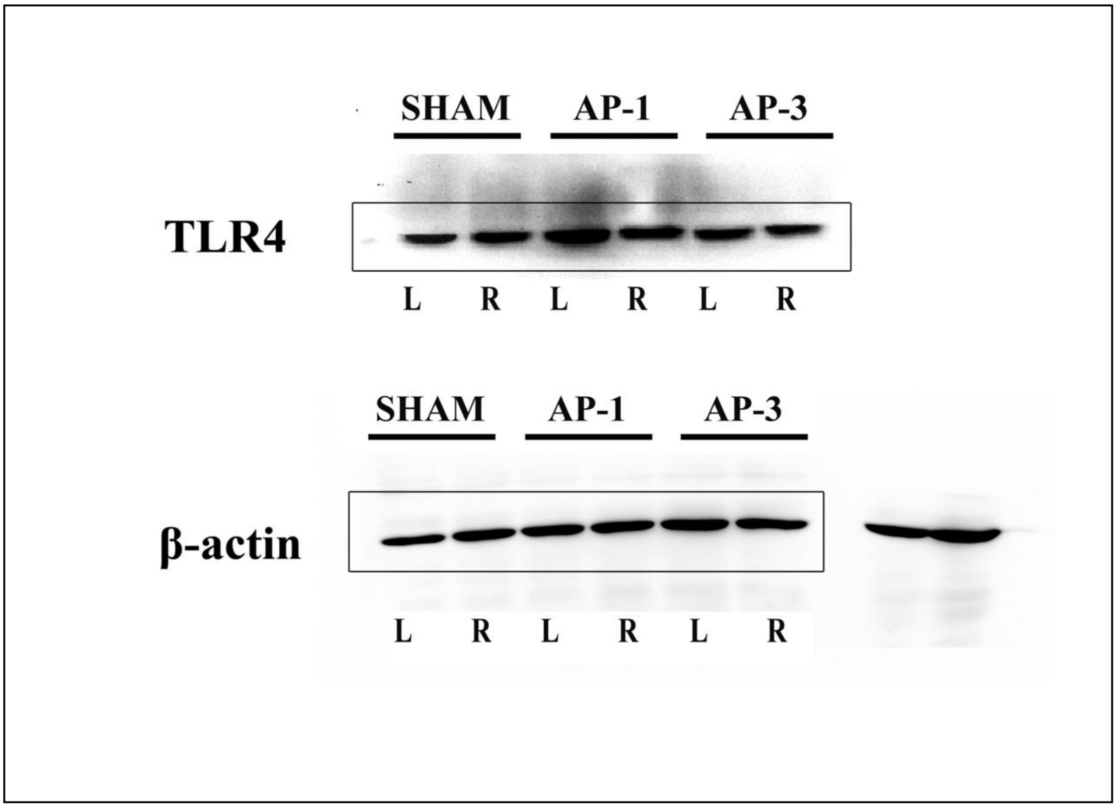

Figure s2 Unedited full-size blots of Supplementary figure 2.

Figure s3:

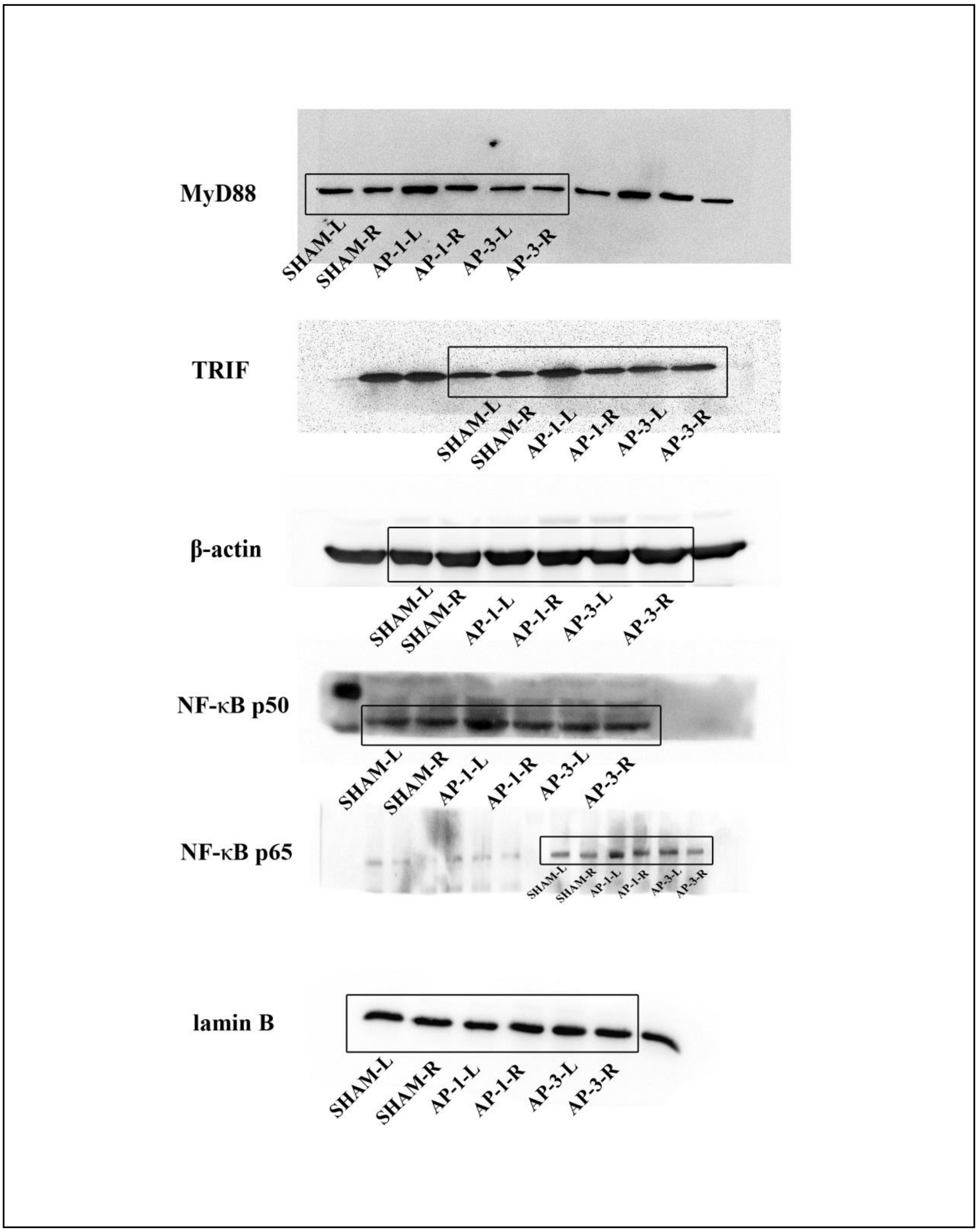

Figure s3 Unedited full-size blots of Supplementary figure 4.

Figure s4:

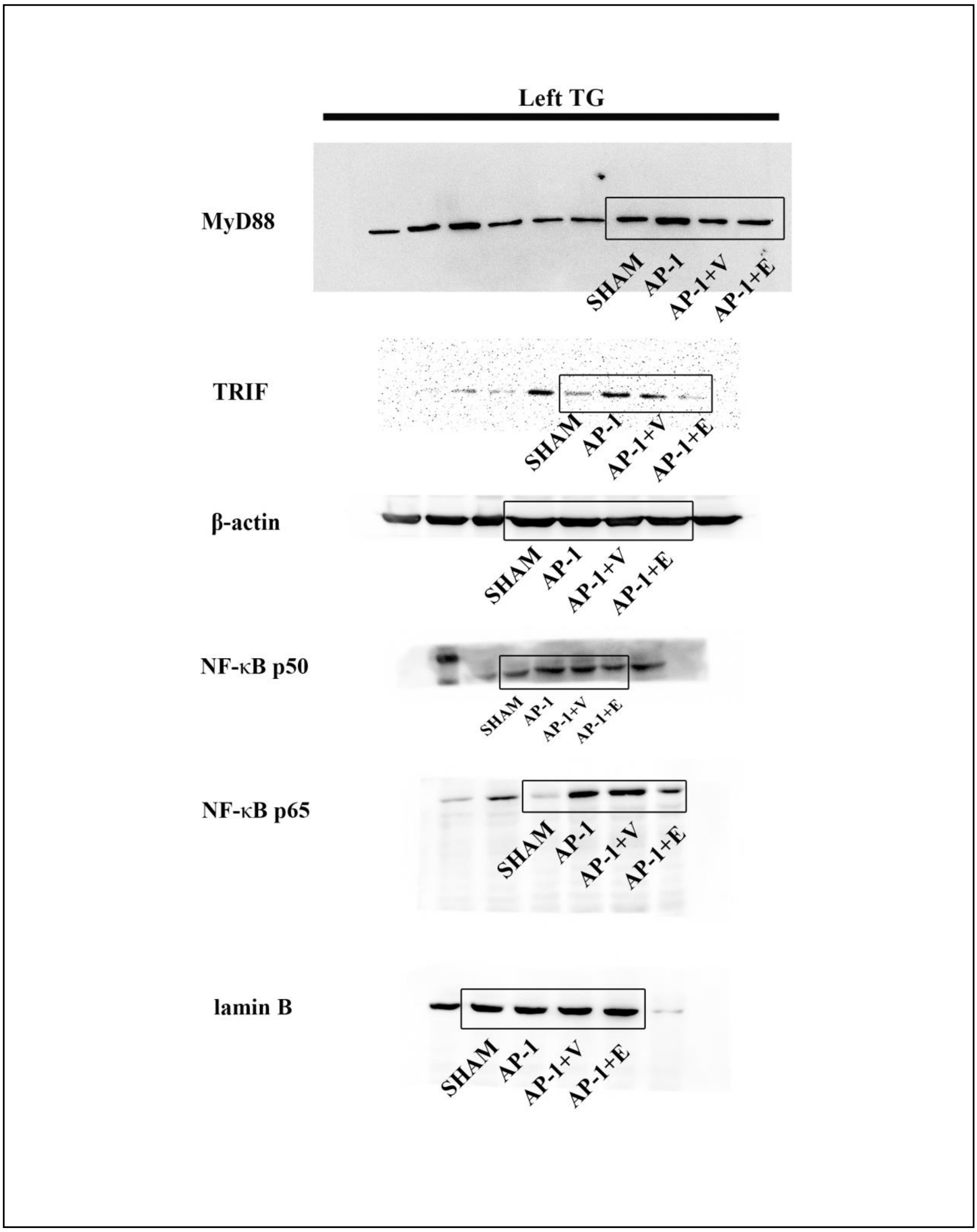

Figure s4 Unedited full-size blots of Supplementary figure 6.
